# Recalls of tattoo and permanent makeup inks in the United States and a follow-up microbiological survey of inks with a previous recall history

**DOI:** 10.3389/fpubh.2023.1279884

**Published:** 2023-11-10

**Authors:** Sunghyun Yoon, Sandeep Kondakala, Mi Sun Moon, Mei-Chiung J. Huang, Goran Periz, Steven L. Foley, Ohgew Kweon, Seong-Jae Kim

**Affiliations:** ^1^Division of Microbiology, National Center for Toxicological Research, U.S. Food and Drug Administration, Jefferson, AR, United States; ^2^Office of Cosmetics and Colors, Center for Food Safety and Applied Nutrition, U.S. Food and Drug Administration, College Park, MD, United States

**Keywords:** tattoo ink recall, microbial contamination, microbiological survey, bacterial identification, US market

## Abstract

In this study, we collected voluntary recall records of tattoo and permanent makeup ink from the U.S. Food and Drug Administration (US FDA) Enforcement Report Database. The recall records contain information, such as recall date, manufacturer, ink color, reason for recall, and the microorganisms detected from the ink samples. Between 2003 and 2021, a total of 15 voluntary tattoo ink recalls occurred in the U.S. market, involving over 200 tattoo inks marketed by 13 manufacturers and one distributor. Fourteen recalls were due to microbial contamination, and one recall was due to allergic reaction. As follow-up, a microbiological survey of 28 tattoo inks of new batches from seven manufacturers having products that were previously recalled was conducted. Aerobic plate count (APC) and enrichment culture methods based on the FDA’s Bacteriological Analytical Manual (BAM) were used to detect microbial contamination. The results revealed that six out of 28 tattoo inks were contaminated with bacteria and were produced by two manufacturers. The level of microbial contamination was less than 250 CFU/g in three of the tattoo inks and between 1 × 10^3^ and 1 × 10^5^ CFU/g in the other three inks. Eleven bacterial isolates were identified, including spore-forming *Bacillus*-related species and potentially pathogenic species. Overall, this study shows that some tattoo ink products produced by manufacturers with a recall history continue to be contaminated with microorganisms. This highlights the need for ongoing monitoring and quality control of such products.

## 1. Introduction

The popularity of tattooing has increased in the United States among adults. Approximately 21 percent of adults in 2012 had at least one tattoo and over 30 percent in 2019 (1, 2). The incidence of tattoo-related complications is also increasing (3–5). Among the various tattoo-related complications reported are microbial infections, and inflammatory or hypersensitivity allergic reactions (6–8). Microbial infections can result from insufficient hygiene practices, such as the use of nonsterile tattoo instruments, or from tattoo inks being or becoming contaminated with pathogenic microorganisms (7, 8). Previously, a series of outbreaks involving *Staphylococcus aureus* and nontuberculous mycobacteria (NTM), including *Mycobacterium chelonae*, a causative agent of skin infection, were linked to contaminated tattoo inks (9–14). Studies have reported that 10–86% of marketed tattoo inks are microbially contaminated, including potentially pathogenic microorganisms (15–21).

Recently, updates on the tattoo ink FDA webpage^1^ revealed that manufacturers conducted 18 voluntary recalls of tattoo inks contaminated with microorganisms between 2003 and 2023 (22). That is, tattoo inks associated with microbial infections are removed from the U.S. market by their manufacturers and distributors. The 2003 recall was the first tattoo ink recall recorded in the U.S. The contaminated tattoo inks associated with the NTM outbreaks in 2012 and 2015 in the U.S. led to nationwide voluntary recalls (12, 13). However, little is known about the current safety of tattoo ink products which are manufactured and/or distributed by facilities that previously underwent voluntary recalls. To our knowledge, no follow-up information is available about the microbial safety of these products.

In this survey, we initially reviewed retrospective recall records of tattoo inks from 2003 to 2021 to retrieve information regarding: how many tattoo ink recalls were issued, reasons for the recalls, including any known illnesses or adverse events associated with recalled products, and by whom and when the problem was initially recognized. Next, we assessed the current microbial contamination in new lots of tattoo inks from facilities that were previously involved in recalls.

## 2. Materials and methods

### 2.1. Information retrieval

Information on recalls of tattoo and permanent makeup (PMU) inks was obtained from US FDA website^2^,” warning letters on recalled tattoo inks, and Enforcement Report database.^3^ The recall information was reviewed to determine the following: the recall year; the manufacturer, brand and ink names; ink color; manufacturer’s country of origin; recall classification Class (I, II, or III) that identifies the degree of health hazard presented by the product; reason for recall; number of inks involved in each recall; the microbes identified (if applicable); and any additional information obtained by the agency, such as who recognized the problem for which the product was recalled, if available.

### 2.2. Ink sampling

We purchased 28 tattoo and PMU inks from seven manufacturers whose products had previously been recalled. We purchased 3–6 bottles of each individual ink with the same lot number, confirmed that the bottle packaging was intact and sealed upon arrival, and stored them in a stainless-steel storage cabinet at room temperature. For each bottle of tattoo ink, we recorded the lot number, ingredients, sterility claim(s), manufacturing location, and expiration date, if available, from the product label or material safety data sheet.

### 2.3. Microbiological analysis

Tattoo inks were analyzed for bacterial and fungal contamination based on the analytical methods described in the FDA’s Bacteriological Analytical Manual (BAM) chapter 23, “Methods for Cosmetics” (23), and BAM chapters 3, “Aerobic Plate Count,” was used for the enumeration of aerobic plate counts (24). Briefly, modified Letheen agar (MLA) and potato dextrose agar (PDA) with chlortetracycline (40 μg mL^−1^) were used for the detection of bacteria and fungi, respectively. Ink samples (1 gram) were serially diluted using modified Letheen broth (MLB) up to 10^−3^. One mL of 10^−1^ dilution was plated on 2 MLA plates (500 μL each) and 100 μL (×2) of each 10^−1^, 10^−2^, and 10^−3^ dilutions were additionally plated on MLA plates. Diluted samples were also enriched for up to 7 days and then streaked (~5 μL) on MLA plates to detect the presence of microorganisms according to BAM Chapter 23. For quality controls, plates and culture media, spiking with and without test microorganisms including *Staphylococcus aureus* (ATCC 25923), *Candida albicans* (ATCC 10231), *Pseudomonas aeruginosa* (ATCC 27853), and *Klebsiella pneumoniae* (ATCC 13883), were analyzed.

### 2.4. Identification of bacterial isolates

Isolates from the original MLA plates were sub-cultured before identity testing via the automated micro-identification system, VITEK 2 Compact System (BioMerieux, Inc., Durham, NC), with GN, GP, and BCL colorimetric cards. These cards identify Gram negatives, Gram positives, and *Bacillus* species, respectively. The inoculum suspension was prepared in 0.45% saline, giving the equivalent of a 0.5 McFarland turbidity. The respective VITEK test cards were filled with the cell suspension according to the manufacturer’s instruction. Sequencing of 16S rRNA genes was also used to identify bacteria using standard methods (25). Briefly, we used a colony PCR amplification with the 16S rRNA gene primers 27f and 1492r (25). PCR products were purified with ExoSAP-IT (USB Corporation, Cleveland, OH), as recommended by the manufacturer. DNA sequences were determined at the sequencing core at the University of Arkansas for Medical Sciences in Little Rock, AR,^4^ and sequence search and comparison was performed using NCBI BLAST.

## 3. Results

### 3.1. U.S. recall records of tattoo inks

A total of 15 voluntary tattoo ink recalls involving 13 manufacturers and one distributor were tracked from 2003 through 2021 (Table 1). One manufacturer, Solid Ink, had two recalls, in 2018 and 2019, respectively. In 2003, Premier Pigments initiated a voluntary recall of five ink colors used in PMU; however, the firm extended its recall to include all affected inks in that line in 2004 due to continuing adverse events submitted to the firm. Premier Pigments products were the PMU inks among recalled inks; all others were tattoo inks. Recalls were not concentrated on any particular ink color, but outbreaks before 2017 were frequently associated with black or gray inks. While in seven instances only a single ink color was recalled, in the other eight instances, multiple ink colors up to 182 inks were recalled. There was no information available on the actual number of ink bottles included in each recall from the U.S. market. Only one recall was conducted for an imported tattoo kit, which included inks and needles (White and Blue Lion in 2014) and testing results showed that both inks and needles were contaminated with multiple microorganisms.

**Table 1:** Information on recalled tattoo and PMU inks from 2003 to 2021.

No.	Year	Manufacturer	Brand	Color	No. of colors	Origin	Reason for recall	Recall Class^a^	Specific reason	Comments
1^b^	2003	Premier Pigments	True Color	5 ink shades	5	Domestic	Adverse skin reaction	I	Benzimidazolone (suspected but not confirmed)	Limited recall initiated based on allergic reaction reports to the firm
	2004	Premier Pigments	True Color	All colors	182	Domestic	Adverse skin reaction	I		All inks from “True Color” line recalled
2	2004	Papillon Studio Supply & Manufacturing Inc.	StarBrite	Black Magic	1	Domestic	Microbial contamination	II	Acremonium mold *Pseudomonas aeruginosa*	Recall initiated by firm
3	2011	Kingpin Tattoo	One	Black	1	Domestic	Microbial contamination	II	*Mycobacterium abscessus* *Mycobacterium chelonae*	Part of 2011–2012 *Mycobacterium* outbreak
4	2012	4 Forty 4 Tattoo	Catfish Carl’s	Grey washes	3	Domestic	Microbial contamination	II	*Mycobacterium chelonae*	Part of 2011–2012 *Mycobacterium* outbreak
5	2014	Minko Inc.	Blacker	Black	1	Domestic	Microbial contamination	II	*Nocardia farcinica* *Nocardia* spp.	
6	2014	White and Blue Lion^c^	White and Blue Lion	All colors	40	Imported	Microbial contamination	II	*Bacillus* spp. *Sphingomonas paucimobilis* *Acinetobacter* spp. *Staphylococcus haemolyticus*	Recall initiated after consumer complaint. FDA sampling/analysis found needles were also contaminated with *Micrococcus luteus, Corynebacterium* spp., and *Clostridium botulinum*, *Clostridium* spp.
7	2015	A Thousand Virgins Corp.	G1, G2, G3	Grey washes	3	Domestic	Microbial contamination	II	*Mycobacterium chelonae* *Microbacterium* sp. *Cryptococcus albidus* *Penicillium* sp.	Recall initiated after Florida state *Mycobacterium* outbreak investigation Three shades of greywash ink recalled
8	2017	Fusion Ink	Fusion Ink	Light Blue Pretty Purple Gamma Green Orange Royal Blue	5	Domestic	Microbial contamination	II	*Oligella ureolytica* *Aeromonas salmonicida* *Pseudomonas andersonii* *Pseudomonas caeni* *Pseudomonas* spp. *Bacillus nealsonii* *Bacillus circulans* *Bacillus horneckiae* *Lysinibacillus sphaericus* *Lysinibacillus fusiformis* *Citrobacter freundii* *Corynebacterium ammoniagenes* *Brevibacillus choshinensis* *Bordetella bronchiseptica*	FDA inspection and survey
9	2017	Radiant Colors	Radiant Colors	Lining Black	1	Domestic	Microbial contamination	II	*Bacillus altitudinis*	FDA survey
10	2018	Solid Ink	Solid Ink	Orange	1	Domestic	Microbial contamination	II	*Bacillus pumilus* *Bacillus licheniformis* *Pseudomonas* sp.	FDA survey
11	2018	Intenze	Intenze	Red Blue	2	Domestic	Microbial contamination	II	*Bacillus halosaccharovorans* *Brachybacterium conglomeratum* *Pseudomonas andersonii* *Pseudomonas balearica*	FDA survey
12	2018	Eternal Ink	Eternal Ink	Blue Red	2	Domestic	Microbial contamination	II	*Bacillus cohnii* *Pseudomonas andersonii* *Lysinibacillus fusiformis*	FDA survey
13	2019	Scalp Aesthetics	Scalp Aesthetics	Basic Black	3	Domestic	Microbial contamination	II	*Pseudomonas aeruginosa* *Brevibacillus choshinensis* *Clostridium butyricum* *Clostridium clostridioforme*	FDA survey High levels of microbes
14	2019	Dynamic Color	Dynamic Color	Black	1	Domestic	Microbial contamination	II	*Bacillus cereus* *Staphylococcus equorum* *Kocuria kristinae*	FDA survey
15	2019	Solid Ink	Solid Ink	Diablo	1	Domestic	Microbial contamination	II	*Clostridium clostridioforme* *Clostridium ramosum* *Bacillus pumilus* *Bacillus megaterium*	FDA survey

^a^21 CFR Part 7, Subpart A, §7.3 (m) Recall classification: (1) Class I is a situation in which there is a reasonable probability that the use of, or exposure to, a violative product will cause serious adverse health consequences or death. (2) Class II is a situation in which use of, or exposure to, a violative product may cause temporary or medically reversible adverse health consequences or where the probability of serious adverse health consequences is remote. (3) Class III is a situation in which use of, or exposure to, a violative product is not likely to cause adverse health consequences.

^b^Premier pigments 2003–2004 recalls counted as one recall. In 2003, the firm initially recalled five ink shades. However, after receiving more consumer complaints, the firm extended their recall to include all affected inks in 2004.

^c^This is the product distributor in the U.S. The product is a self tattoo kit having inks and needles.

#### 3.1.1. Reasons for recalls

The FDA records showed that six of the seven recalls that occurred prior to 2015 were triggered by microbial infection or adverse skin reactions, reported by consumers to manufacturers or regulatory authorities (i.e., states and FDA). Since then, recalls were initiated as a result of FDA’s surveillance and inspection programs (eight recalls) find microorganism contaminated inks available on the U.S. market. This effort led to voluntary recalls by the firm. Most voluntary recalls were due to microbial contamination but the recall of ink products from Premier Pigments was initiated due to allergic reactions reported to the firm. Among recalls associated with microbial contamination, three recalls involved outbreaks of NTM skin infections in multiple states, including New York, Washington, Iowa, Colorado, and Florida, during 2011–2012 and 2015. All but one recall was assessed as Class II, indicating that products may cause temporary or reversible adverse health consequences or where the risk of serious harm is remote. One recall (Premier Pigments ink products) was assessed as Class I, the most serious class with a reasonable probability of causing serious adverse health outcomes or death (26). The recall was initiated due to reports of adverse events related to the ink products, which caused swelling, cracking, peeling, blistering, scarring, and granuloma formation. In some instances, the adverse reactions resulted in serious disfigurement, leading to difficulties with eating and speaking (27).

#### 3.1.2. Microorganisms detected from the recalled inks

The 15 recalls identified 51 microorganisms, including 48 bacteria and three fungi (Table 1). The bacterial isolates belonged to 18 genera and 33 species. The genus *Bacillus* was the most prevalent (12 isolates, 24%). When the genera *Brevibacillus* (2 isolates) and *Lysinibacillus* (2 isolates) were included, spore-forming bacillus-related isolates accounted for nearly 30% of the total (16 isolates). The next two most common isolates belonged to the genera *Pseudomonas* (9 isolates, 18%) and *Mycobacterium* (4 isolates, 8%). The fungi were identified as *Acremonium*, *Cryptococcus*, and *Penicillium*.

### 3.2. Microbiological survey of tattoo inks

We evaluated microbial contamination in 28 sealed tattoo and PMU inks from seven manufacturers whose products had previously been recalled (Table 2). Tattoo ink product labels from five manufacturers (#2, #3, #4, #5, and #6) claimed the products were sterile; whereas sterility claims for the inks from manufacturers #1 and #7 were not available. Overall APC and enrichment culture analysis revealed that six inks (21%) showed microbial contamination: three inks each from manufacturer #1 and manufacturer #4 (Table 2). No microbial contamination was detected in the tested ink products from the other five manufacturers. Total microbial counts were below 250 CFU per gram in three ink samples and higher than 1 × 10^3^ CFU per gram in another three ink samples, with the highest count being 2.5 × 10^6^ CFU per gram. Eleven bacterial species, including 5 species belonging to *Bacillus*, were identified. Possibly pathogenic bacteria, such as *Kocuria rosea*, *Aerococcus viridans*, and *Alloiococcus otitis*, were identified (Table 2). No fungi were detected.

**Table 2:** Culture-based detection of microorganisms from tattoo inks.

Manufacturer	Ink	Claim sterility^a^	CFU/gram^b^	Identified microorganisms
1	1	N	<10	
	2	N	<10	
	3	N	<250	*Bacillus clausii*
	4	N	<10	
	5	N	<250	*Bacillus smithii*
	6	N	<250	*Oceanobacillus iheyensis*
				*Bacillus firmus*
	7	N	<10	
	8	N	<10	
2	9	Y	<10	
	10	Y	<10	
3	11	Y	<10	
	12	Y	<10	
	13	Y	<10	
	14	Y	<10	
	15	Y	<10	
4	16	Y	<10	
	17	Y	6.5 × 10^5^	*Bacillus cereus*
				*Kocuria rosea*
	18	Y	1.1 × 10^3^	*Bacillus clausii*
				*Alkalihalobacillus halodurans*
				*Alicyclobacillus acidocaldarius*
	19	Y	<10	
	20	Y	<10	
	21	Y	5.4 × 10^5^	*Aerococcus viridans*
				*Alloiococcus otitis*
5	22	Y	<10	
	23	Y	<10	
	24	Y	<10	
6	25	Y	<10	
	26	Y	<10	
7	27	N	<10	
	28	N	<10	

^a^Y for yes if a sterility claim was made on the label, and N for no if no sterility claim was made.

^b^<10, if no colonies were obtained; <250, if colony count was <25 at 10^−1^ dilution.

## 4. Discussion

In recent years, the popularity of tattoos has increased, resulting in a corresponding increase in tattoo-related complications. According to a 2015 online poll, 29% of adults in the U.S. reported having a tattoo. This marked a substantial increase from 21% in 2012, 16% in 2008, and 14% in 2003 (28). Along with this trend, the U.S. FDA has observed an increase in the number of tattoo ink recalls between 2003 and 2023. In total, 18 voluntary recalls have been recorded in the U.S. market ((22); a), commencing with the initial recalls in 2003 and 2004, prompted by adverse events reported by consumers (29). Of note, in the time frame of interest in this survey, 2003–2021, there were 15 voluntary recalls. More than half of these (8 recalls) occurring from 2017 to 2021 were prompted by the FDA’s surveillance programs, that included sampling and microbiological analysis of tattoo inks, and were followed by voluntary recalls by manufacturers. The results of this surveillance efforts highlight the ongoing efforts to address the safety and quality of tattoo ink products and the importance of this activity to reduce the risk to consumers (30).

The recall records show that there are two main reasons for recall: microbial contamination and allergic reactions. In 14 out of the 15 recall cases, the main reason was associated with microbial contamination of tattoo ink. One recall was related to allergic reactions, but the causative agent was not confirmed.

The recall records provide taxonomical identification of microbial contaminants found in tattoo inks which can help identify potential sources of contamination and improve surveillance and preventive programs. The recall records have shown that *Bacillus* species and other spore-forming bacilli are the most commonly identified groups of microbial contaminants. This is consistent with the findings of previous microbial surveys of tattoo inks (15–21).

As shown in this study, 6 out of 28 ink samples, from two of the seven manufacturers’ inks samples demonstrated microbial contamination. These results indicate that despite previous surveys and publications reporting this problem since 2004 (31), microbial contamination continues to be an issue. These results also emphasize the importance of follow-up monitoring of the manufacturers of tattoo inks.

In conclusion, we analyzed tattoo ink recall records spanning almost 20 years. Microbial contamination was the leading cause of voluntary tattoo ink recalls in the U.S. According to our microbiological survey results, some tattoo ink products from manufactures with a previous recall history were still contaminated with microorganism. These findings emphasize the importance of raising consumer awareness about the public health and safety of tattoo inks, as well as the continued monitoring of tattoo ink manufacturers having previous recalls.

## Data availability statement

The original contributions presented in the study are included in the article/supplementary material, further inquiries can be directed to the corresponding author.

## Author contributions

SY: Investigation, Writing – original draft. SK: Investigation, Writing – original draft. MM: Investigation, Resources, Writing – review & editing. M-CH: Writing – review & editing. GP: Writing – review & editing. SF: Writing – review & editing. OK: Conceptualization, Supervision, Writing – review & editing. S-JK: Conceptualization, Supervision, Writing – review & editing.

## Footnotes

^1^ https://www.fda.gov/cosmetics/cosmetics-news-events/fda-issues-draft-guidance-tattoo-inks

^2^ https://www.fda.gov/cosmetics/cosmetic-products/tattoos-temporary-tattoos-permanent-makeup

^3^ https://www.accessdata.fda.gov/scripts/ires/index.cfm#tabNav_advancedSearch

^4^ http://mbim.uams.edu/research-cores/dna-sequencing-core-facility
